# Examination of Entire Gastrointestinal Tract: A Perspective of Mouth to Anus (M2A) Capsule Endoscopy

**DOI:** 10.3390/diagnostics11081367

**Published:** 2021-07-29

**Authors:** Ji Hyung Nam, Kwang Hoon Lee, Yun Jeong Lim

**Affiliations:** 1 Division of Gastroenterology, Department of Internal Medicine, Dongguk University Ilsan Hospital, Dongguk University College of Medicine, Goyang 10326, Korea; drnamesl@gmail.com; 2 Division of Rheumatology, Department of Internal Medicine, Dongguk University Ilsan Hospital, Dongguk University College of Medicine, Goyang 10326, Korea; lkh24217@hanmail.net

**Keywords:** active locomotion, capsule endoscopy, gastrointestinal tract, M2A capsule, pan-endoscopy

## Abstract

Capsule endoscopy (CE) is the only non-invasive diagnostic tool that enables the direct visualization of the gastrointestinal (GI) tract. Even though CE was initially developed for small-bowel investigation, its clinical application is expanding, and technological advances continue. The final iteration of CE will be a mouth to anus (M2A) capsule that investigates the entire GI tract by the ingestion of a single capsule. This narrative review describes the current developmental status of CE and discusses the possibility of realizing an M2A capsule and what needs to be overcome in the future.

## 1. Introduction

Capsule endoscopy (CE) involves an electro-optical device that captures and transmits images inside the gastrointestinal (GI) tract while a compact wireless capsule travels to the distal part after being swallowed. The CE system includes three main components, an ingestible wireless capsule, data recorder, and computer for software analysis. It is less invasive and convenient and does not require sedation, making it safe and comfortable to examine patients who failed or declined conventional endoscopic examinations. The first CE device was originally developed as a mouth to anus (M2A) capsule [1]. As the name implies, the capsule is like an oral pill used to examine the entire GI tract as it travels from the mouth to the anus. However, CE using that capsule lacked movement and orientation control and subsequently had suboptimal diagnostic accuracy compared to conventional endoscopy, so it has mainly been applied to small-bowel examinations inaccessible by conventional endoscopes, and was renamed the PillCam SB (Given Imaging, Yoqneam, Israel) [1]. Nevertheless, clinical applications are expanding to other segments of the GI tract. Diverse wireless CEs have been developed for imaging the colon, esophagus, and stomach, and technological advances continue to investigate the entire GI tract. Capsules for the GI tract other than the small bowel are already commercially available in many countries. To overcome the limitations regarding uncontrolled CE motion, various technical attempts including tethering, magnetic control, and internal locomotion are being made [2,3,4]. However, it is not easy to develop an M2A capsule because the complex functions that need to be built inside the capsule vary for each part of the GI tract. To realize an M2A capsule, several challenges must be overcome, such as staying in the esophagus against gravity and peristalsis, controlling movement and increasing the resolution in the wide gastric cavity, passing through the pylorus and lowering gastric transit time, bowel preparation through the long length of the intestine, and maintaining its operation until capsule excretion. These problems have already been resolved considerably in CE for each GI tract segment, including magnetic control of the capsule, modifying the frame rate, bidirectional view, improvement in resolution, recognition of location, a booster administration of bowel-cleansing agent, and lengthening the battery time. In light of the recent advances, CE has the potential to offer a perfect overview of the entire GI mucosa. In this review, we summarize the current status of technological advances from the perspective of the era in which pan-endoscopy is possible and discuss the feasibility of real M2A capsule endoscopy in the future and what needs to be overcome for use in clinical practice. 

## 2. Esophageal Capsule Endoscopy

The key to the clinical application of esophageal CE (ECE) is how long it can stay in the short and straight esophageal lumen to obtain sufficient images. Therefore, not only improving the integrated capability of the capsule but intentional control of the capsule location through patient posture or tethering have been reported in the literature and real practice. Since the first ECE (PillCam ESO; Given Imaging, Yoqneam, Israel) was approved by the Food and Drug Administration (FDA) in 2004 [5], PillCam ESO2, as the second-generation ECE, has become clinically available (Table 1). While ECEs are similar to small-bowel CE (SBCE) devices in size (11 × 26 mm), the integrated features have been technically modified to account for the fast passage through the esophagus by using a short operation time (20–30 min), a bidirectional capsule (dual cameras with one on each end), a wide angle, and a high image capture rate. To effectively observe the esophagus, patients are instructed to follow a standard protocol for ECE [6]: fasting for over two hours, ingestion of the capsule in a supine position, and recording during gradual tilting from supine to 30° then 60°, followed by an upright position. This is to obtain images evenly throughout the esophagus. However, postural control alone may not allow sufficient esophageal transit time, especially in the upper esophagus. A randomized controlled trial proved that magnetically controlled ECE stayed longer in the esophagus than the original PillCam ESO2 [7]. However, the magnetic forces were not strong enough to hold the capsule against peristalsis near the gastroesophageal junction (GEJ). In addition, increased magnetic force or tilting and rotation of the capsule within the esophagus can cause patient discomfort related to retrosternal pressure. Additionally, because regurgitation of the capsule during manipulation in the upper esophagus can increase the risk of aspiration, especially in the head-down position, clinical applications must proceed cautiously for elderly or disabled patients.

Several studies have been conducted on the diagnostic accuracy of ECE. The Z-line was visualized in 90% of patients who swallowed the capsule in the right supine position [8]. Esophageal pathology is most commonly presented at the distal esophagus near the Z-line. Hence, the current indications for ECE focus on Barrett’s esophagus (BE) and esophageal varix. As the capsule rapidly passes the upper esophagus by swallowing and stays for a relatively long time in the lower esophagus before passing the GEJ, lesions in the GEJ or distal esophagus can be easily detected under adequate patient posture. In meta-analyses primarily using PillCam ESO (or ESO2), ECE was safe and preferred by patients, but the diagnostic accuracy for BE or esophageal varix using traditional esophagogastroduodenoscopy (EGD) as a reference standard was limited [9,10]. External controls using magnets or strings may improve the operability in the upper GI (UGI) tract, which will be introduced in the next section. 

The modified PillCam ESO2 identified gross blood in the UGI tract significantly more often than nasogastric tube aspiration in an emergency setting [11]. On this basis, the third-generation PillCam ESO3 (called PillCam UGI) was released [12], which was further enhanced to obtain more images through the entire UGI tract. The capsule is intended for visualization of the UGI tract and is specially designed to detect gross blood. Dual frame rate technology operates at 35 frames per second (fps) for the first 10 min considering the esophageal location and 18 fps for the last 80 min for the stomach. This system may be used for hemodynamically stable patients with suspected UGI bleeding. Earlier this year, a new UGI capsule, the MiroCam MC2400 (Intromedic, Seoul, Korea), was approved by the Communauté Européenne Medical Devices Directive (CE MDD, 289147-2019-CE-KOR-NA-PS Rev. 1.0, 8 January 2021) and will be commercially available soon. It also has two cameras, one at each end, and obtains images at 24 (12 × 2) fps at a 170° angle of view. It is slightly longer (10.8 × 30.1 mm) than the small-bowel MiroCam capsules and it operates for 90 min. As for the other ECE technologies, tethered CE can safely evaluate the microscopic structure of the esophagus without endoscopic assistance [13,14,15]. Additionally, the esophageal endoscopic system combined with a universal serial bus (USB) is not limited by battery time [16]. The device is routed through the USB port to the computer and controlled by the length of the swallowed cable. Even though the capsules used in these experiments are not strictly wireless, they may be considered a technical background for pan-endoscopy.

## 3. Gastric Investigation via Active Locomotion

As the CE device is passively moved by peristalsis and gravity, there is a possibility of incomplete inspection in the wide lumen of the stomach. Conventional EGD currently appears to be an irreplaceable method for gastric examination. However, considering the discomfort and invasiveness of EGD, non-invasive screening using wireless CE cannot be abandoned. Thus, many trials for the active locomotion of CE have been conducted. Initially, interest was focused on internal locomotion that does not require magnetic fields and auxiliary equipment. Active propulsion of the capsule can improve CE performance in the stomach and reduce gastric transit time. Diverse technologies for internal locomotion including electrode-stimulated muscle contraction, inchworm-like mechanisms, crawling by legs, and hydrodynamic force-based mechanisms such as a propeller have been attempted [17,18]. However, the results were unsatisfactory and a CE device capable of internal locomotion failed to be commercialized. The main problems were high power consumption and limited capsule volume, making it difficult to integrate advanced technologies and store energy.

**Figure 1:**
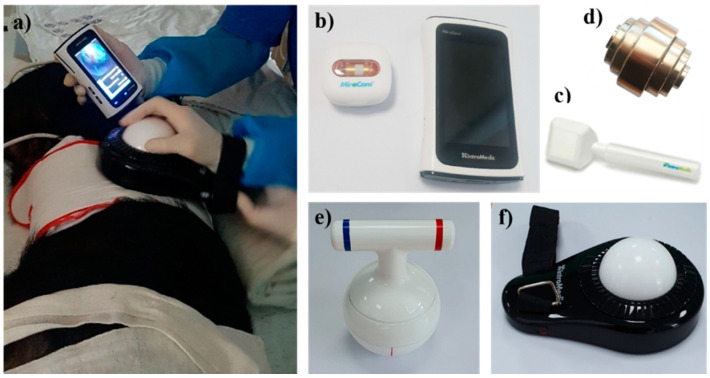
Magnetically assisted capsule endoscopy. (**a**) A preclinical study using hand-held controllers; (**b**) MiroCam MC4000-M with a real-time receiver; various hand-held controllers including (**c**) hammer shaped, (**d**) stack shaped, (**e**) kettlebell type, (**f**) track ball type.

In contrast, external control using magnetic force is free from capsule capacity and power limitations [2]. The first study on human gastric examination by CE with magnetic force (using a modified PillCam COLON with a hand-held magnet) was published in 2010 [19]. Subsequently, the remote control of a magnetic CE device was considered safe and clinically feasible in healthy volunteers [20]. Another magnetically assisted CE (MACE) device using a hammer-shaped hand-held controller (MiroCam Navi System, Intromedic, Seoul, Korea) was developed and showed good maneuverability and high diagnostic accuracy for gastric investigation [21,22]. The device can control the location and orientation of the capsule while allowing viewing of the video directly through a real-time receiver. This MiroCam Navi also presented high diagnostic accuracy for BE (sensitivity 94% and specificity 100%) in a pilot study [23]. Recently, we conducted a preclinical study on various hand-held controllers that are more easily manipulated (Figure 1), which will be tested for safety and tolerability in further human studies. The hand-held controllers are easy to operate with simple training, so they may replace conventional EGD after guidelines on how to use the controller have been established. Recently, a detachable string magnetically controlled CE (DS-MCCE) system (Ankon Technologies, Shanghai, China) was introduced, and it was feasible and well-tolerated for the detection of esophageal or gastric cardiac lesions [24,25]. Meanwhile, CE with an automatic robotic control system was first attempted in China. Since a pilot study was published in 2012 [26], ESNavi (Ankon Technologies, Shanghai, China), a magnetically controlled capsule gastroscopy (MCCG) with a robotic control system, was proven safe and feasible for largescale population-based gastric cancer screening [27]. It is currently commercially available in China. Several comparative studies of MCCG versus traditional EGD showed good diagnostic yield for gastric lesions, supporting that MCCG has a benefit as an alternative to conventional EGD [28,29,30]. Although the large volume of the equipment remains a challenge, population-based gastric cancer screening by the MCCG is clinically meaningful in high-prevalence areas.

The magnetic locomotion capabilities using hand-held controllers or a robotic system have yielded excellent results as a tool for gastric examination, but such capabilities are useless in the lower GI tract. When applied to the M2A capsule, the role of navigation by external locomotion is only until the capsule passes through the pylorus. Considering a real M2A capsule that only needs to be swallowed without external control, an optimal gastric view and pyloric passage are critical issues. Deforming agents such as simethicone and antimotility drugs to reduce peristalsis help to optimize the gastric view [31]. However, prolonged gastric transit time by these medications can interfere with capsule excretion within the operation time. To completely substitute for the role of conventional EGD, the capsule should pass through the pylorus and reach the ampulla of Vater (AoV). As the time interval between the pylorus and the AoV varies widely, from only seconds to more than an hour, the view of the duodenal bulb and the second part of duodenum can be hampered or overexposed. 

## 4. Small-Bowel Capsule Endoscopy

Capsule endoscopy is currently the most preferred and widely used diagnostic method for the investigation of small-bowel diseases. In the ESGE clinical guidelines, the indications for SBCE are clearly described as obscure GI bleeding (OGIB), iron deficiency anemia, known or suspected Crohn’s disease (CD), small-bowel tumors, inherited polyposis syndromes, abnormal radiological imaging, and subgroups of celiac disease [32]. Performance measures and technical reviews for SBCE quality control have also been proposed in detail and were recently updated [32,33]. The first CE was developed in 2001 (PillCam SB; Given Imaging, Yoqneam, Israel), and now many SBCE devices have been released and are currently in use (Table 1).

After the M2A capsule passes through the pylorus, the transit time and excretion of the capsule are the key issues. Since the SBCE devices developed to date cannot be actively driven, there is no way to reduce the transit time of the capsules in the intestine other than by administering a booster cleansing agent. The PillCam SB3 (Medtronic, Minneapolis, MN, USA) has an adaptive frame rate function, enabling regulation of the image capture rate by a capsule progression speed from 2 to 6 fps. Additionally, the receiver system uses advanced software to skip similar images. This autonomic mode resulted in a reduction in reading time by 50% without compromising the diagnostic rate [34], and was applied from the PillCam SB2 model onward. Endocapsule 10 (Olympus, Tokyo, Japan) is also a widely available SBCE device. The recently developed MiroCam MC4000 (Intromedic, Seoul, Korea) has two lenses at one side of the capsule and can perform size measurements and hardware-enabled 3D reconstruction using images from the two lenses, which is expected to be useful in the characterization of subepithelial lesions [35]. In addition, the resolution was improved compared to conventional 2D CE by using de-noising and de-blurring. CapsoCam (Capso-Vision, Saratoga, CA, USA) is characterized by a wire-free storage design. As it can store images in the capsule, there is no need for external receiver equipment and data transmission. In addition, four lateral onboard cameras that capture a 360° panoramic view provide a detailed examination of the mucosal surface. The second-generation CapsoCam SV-1 detected more cases of bleeding than the PillCam SB3 in patients with OGIB, and the relevant bleeding sources were equally visualized by both CE devices [36]. One of the newest SBCE devices is the OMOM Capsule 2 (Jinshan Science & Technology, Chongqing, China). The OMOM2 and PillCam SB3 had a similar diagnostic yield for OGIB patients in a randomized comparative study [37].

## 5. Colon Capsule Endoscopy

There is no doubt of the importance of colon polyp surveillance and colorectal cancer (CRC) screening for the reduction of CRC mortality. However, colonoscopy is not universally tolerated in relation to its cost, invasiveness, inconvenience, sedation-related risks, and the requirement for an experienced endoscopist. Thus, CE is considered an alternative for conventional colonoscopy in many clinical settings. Additionally, a non-visualized colon segment in previous incomplete colonoscopy can be seen in CE [31,38]. The main issues with colon CE (CCE) relate to incomplete capsule passage and inadequate bowel preparation. To achieve adequate bowel preparation and capsule excretion, CCE requires more extensive bowel cleansing than conventional colonoscopy [39]. Applying CCE in daily practice has shown satisfactory performance and technical improvement [39]. In a prospective colon capsule registry trial, the polyp detection rate was 32% among 161 cases, in which 15% were non-diminutive, exceeding 6 mm [40]. Provided that individuals at high risk of capsule retention can be excluded, CCE can play a role as the first modality in determining the need for colonoscopy.

The first colon capsule (PillCam COLON; Given Imaging, Yoqneam, Israel) was released in 2006 but is no longer in use. Afterward, the second-generation CCE-2 (PillCam COLON2; Medtronic, Minneapolis, MN, USA) was developed (Table 1), with the technological advance of frame rate modification according to the capsule’s location and motion. It is currently available in the United States, Japan, and many European countries. The first key to CCE is the visualization of the entire colonic mucosa, which is related to bowel cleansing and the capsule excretion time [41]. Purgative bowel preparations approved for conventional colonoscopy enable appropriate bowel cleaning for CCE. A large volume of polyethylene glycol has been the most widely used purgative [42]. Sodium phosphate, magnesium citrate, oral sulfate solution, or diatrizoate are used as effective boosters [43,44,45]. As the battery time of the second model is about 10 h, a booster administration of the bowel preparation regimen is required to facilitate capsule excretion. To conserve battery, the CCE-2 itself maintains a sleep mode with under 14 frames per minute while the capsule is in the stomach. Data Recorder 3 of the CCE-2 recognizes when the capsule passes the pylorus into the small bowel, then instructs it to increase the frame rate [46]. Since the colon has circular mucosal folds and a relatively wide lumen, there may be limitations due to dynamic uncontrolled movement. To overcome the blind area, the CCE-2 has two high-resolution cameras at either end, with a 172° angle of view, allowing 344° of visual coverage. The CCE-2 implements an adaptive frame rate from 4 to 35 frames for intestinal investigation. Given that the PillCam SB3′s adaptive frame rate ranges from 2 to 6 fps, the CCE-2 is designed to obtain more images while in rapid motion. The second issue is the diagnostic accuracy of CCE. In a recent meta-analysis involving 1898 patients in 12 studies, the specificity of CCE for polyp detection was 0.85, which increased to 0.88 and 0.95, when cutoffs of 6 mm and 10 mm, respectively, were applied [47]. The relatively low diagnostic accuracy compared to colonoscopy limits the role of CCE as an alternative for CRC screening and is a remaining challenge to be overcome for pan-endoscopy. Image enhancement tools, as well as size measurements, have already been supplied in the reading software [48]. However, further advances toward high image resolution are required to increase the diagnostic accuracy for polyps, especially for <10 mm polyps. When detecting a significant lesion that requires colonoscopy in CCE, it is necessary to locate it. Localization of colorectal lesions based on feature point tracking in images was introduced in a recent study [49]. The authors proposed a model to reconstruct the colon as viewed by the capsule. Positioning of the capsule and reconstruction of the hollow organ by the endoscopic view are necessary for the realization of the M2A capsule. Meanwhile, PillCam Crohn’s (Medtronic, Minneapolis, MN, USA) was designed to observe both the small bowel and colon in patients with CD [50]. This model is similar in size and hardware to the CCE, with two cameras (one on each end), a widely adjusting frame rate (4–35 fps), and a wide-angle view (168° each end). A novel pan-enteric score using this capsule showed excellent reliability in the evaluation of inflammation in CD [51]. Recently, a new CCE device, C-Scan (Check-Cap, Isfiya, Israel), was developed for colon polyp screening, which scans the inner lining of the colon using low-dose X-ray beams (https://check-cap.com/the-c-scan-system/ (accessed on 1 July 2021)). Commercializing CCE devices has important implications for realizing M2A devices because it showed that a capsule swallowed by mouth could reach the end of the GI tract within the operating time.

## 6. Current Trials of M2A Capsules

This paper focuses on the hardware evolution of CE. Mouth to anus CE should be convenient and non-invasive. M2A capsules promise to be an excellent method for GI screening comparable to conventional endoscopy. Various capsules customized for each part of the GI tract have been developed. Several UGI capsules capable of esophageal or gastric screening, and LGI capsules for small- and large-bowel examinations, are already commercialized. At this time, attempts are being made to expand the observation fields using these capsules. Recently, several studies have supported the possibility of M2A screening for the entire GI tract. In 462 asymptomatic 50- to 75-year-olds, whole-GI tract examination using CCE showed a 12% prevalence of clinically relevant abnormalities including severe reflux esophagitis, large polyps, severe ulcers, and CRC [52]. As for the patients with suspected CD, the diagnostic accuracy for erosion or ulcers using CCE-2 in both the small and large bowel was approximately 80%, based on device-assisted endoscopy (DAE) as standard [53]. CE has a similar polyp or cancer detection rate to DAE or conventional colonoscopy [54,55]. The demonstrated diagnostic yield for polyps or cancer supports the utility of M2A capsules for routine surveillance of high-risk patients with inherited intestinal polyposis, such as familial adenomatous polyposis or Peutz–Jeghers syndrome. Among the currently widely used CE devices, COLON2 and Crohn’s capsule are the most applicable products for pan-endoscopy. Here, the technical integration of external control attempted with UGI capsules such as MC4000 and MCCG is practically plausible.

Indeed, it may be inappropriate to mention the M2A capsule, except for the issues of internal locomotion. However, developing a small capsule that incorporates a miniaturized power supply and an internal locomotion system is challenging under the current technology. Considering the passively moving CE devices that are currently in use, the basic requirements of an M2A capsule are, first of all, sufficient battery time to investigate the entire GI tract and a wide field of view via bidirectional lenses. That is why CCE devices are mainly used as a challenge to pan-endoscopy. Another important issue is the range of the adapting frame rate. Staying, moving, and the subsequent transit time of CE devices depend upon complex morphological and physiological differences in the GI tract. Thus, modifying the frame rate over a wide range according to capsule motion can help in obtaining images evenly throughout the GI tract. Under the current status of CE technology, magnetically assisted pan-endoscopy will be possible. After the CE device actively inspects the UGI tract and passes through the pyloric ring by external magnetic control, the capsule can passively move and capture images of the small bowel and colorectum. A booster administration of the bowel-cleansing agent may promote capsule excretion. By artificial intelligence (AI)-based image learning of each GI segment, it may be possible to determine where the capsule is and modify the frame rate. AI could also help complete M2A capsules, including landmark identification, lesion detection, and interpretation. In addition, other imaging modalities, which are currently under investigation, may highlight the expectations for CE as a pan-endoscopy. Several experimental trials for fluorescence-based CE systems have been recently introduced in the literature [56,57,58]. After creating a robust M2A capsule, the application of targeted fluorescence imaging technology enables the biological detection of GI abnormalities by conjugating fluorescent dyes to lesion-specific proteins, which will considerably enhance the functionalities of M2A capsules as a screening tool.

## 7. Conclusions

To implement an M2A capsule, a detailed examination of the entire GI tract without a blind area is essential. This is made possible by controlling the capsule movement. Magnetically controlled bidirectional CCE is currently the most realistic method for pan-endoscopy and has proven its safety and feasibility in many studies. Even though it is still not a convenient test requiring taking only an oral pill in daily practice, it may be a transitional step toward the realization of M2A examinations. The final goal in the field of CE is to develop an actively moving M2A capsule that only needs to be swallowed. Someday, technological innovations will enable pan-endoscopy by micro-miniature robotic capsules with the integration of an internal locomotion device. It would be able to locate and orient itself, move with active propulsion, find significant lesions, and capture images throughout the entire GI tract. AI may help complete M2A capsules, including landmark identification, lesion detection, and interpretation.

## Figures and Tables

**Table 1:** Recent and upcoming models of capsule endoscopy, from esophagus to colon.

	PillCam ESO2	PillCam ESO3 (UGI)	MiroCam MC2400	ANKON NaviVam	PillCam SB3	MiroCam MC4000	Endocapsule 10	CapsoCam SV-1	OMOM Capsule2	PillCam Crohn’s	PillCam COLON2
Manufacturer	Medtronic, USA	Medtronic, USA	Intromedic, Korea	ANKON Technologies, China	Medtronic, USA	Intromedic, Korea	Olympus, Japan	Capso-Vision, USA	Jinshan Science & Technology, China	Medtronic, USA	Medtronic, USA
Size (mm)	11.4 × 26.4	11.6 × 32.3	10.8 × 30.1	11.8 × 27	11.4 × 26.4	10.8 × 24.5	11 × 26	11 × 31	11 × 25.4	11.6 × 32.3	11.6 × 32.3
Weight (g)	2.9	3.0	3.5	4.8	3.0	3.4	3.3	3.8	4.5	3.0	3.0
Number of cameras	2	2	2	1	1	2 (at one end)	1	4	1	2	2
Angle of view	169° ^†^	172° ^†^	170° ^†^	151°	156°	170°	160°	360°	165°	168° ^†^	172° ^†^
Frame rate (fps *)	19	35 ~18 ^‡^	24	2	2–6	2 × 2	2	12–20	2	4–35	4–35
Battery life (hr)	30	90	90	8	12	10	12	15	10	10	10
Data transmission	Radiofrequency	Radiofrequency	Human body communication	Radiofrequency	Radiofrequency	Human body communication	Radiofrequency	Not applicable	Radiofrequency	Radiofrequency	Radiofrequency

* fps, frames per second; ^†^ per end; ^‡^ 35 fps during first 10 min, and 18 fps during next 80 min.

## Data Availability

Not applicable.
